# Radiation therapy after radical prostatectomy is associated with higher other-cause mortality

**DOI:** 10.1007/s10552-022-01564-z

**Published:** 2022-03-01

**Authors:** Christoph Würnschimmel, Mike Wenzel, Francesco Chierigo, Rocco Simone Flammia, Benedikt Horlemann, Zhe Tian, Fred Saad, Alberto Briganti, Sharokh F. Shariat, Michele Gallucci, Nazareno Suardi, Felix K. H. Chun, Derya Tilki, Markus Graefen, Pierre I. Karakiewicz

**Affiliations:** 1 Martini-Klinik Prostate Cancer Center, University Hospital Hamburg-Eppendorf, Martinistraße 52, 20246 Hamburg, Germany; 2 Cancer Prognostics and Health Outcomes Unit, Division of Urology, University of Montréal Health Center, Montreal, QC Canada; 3 Department of Urology, Lucerne Cantonal Hospital, Lucerne, Switzerland; 4 Department of Urology, University Hospital Frankfurt, Frankfurt, Germany; 5 Department of Urology, Policlinico San Martino Hospital, University of Genova, Genova, Italy; 6 Department of Maternal-Child and Urological Sciences, Umberto I Hospital, Sapienza Rome University, Policlinico, Rome, Italy; 7 Department of Urology and Division of Experimental Oncology, URI, Urological Research Institute, IRCCS San Raffaele Scientific Institute, Milan, Italy; 8 Department of Urology, Comprehensive Cancer Center, Medical University of Vienna, Vienna, Austria; 9 Departments of Urology, Weill Cornell Medical College, New York, NY USA; 10 Department of Urology, University of Texas Southwestern, Dallas, TX USA; 11 Department of Urology, Second Faculty of Medicine, Charles University, Prague, Czech Republic; 12 Institute for Urology and Reproductive Health, I.M. Sechenov First Moscow State Medical University, Moscow, Russia; 13 Division of Urology,, Department of Special Surgery, Jordan University Hospital, The University of Jordan, Amman, Jordan; 14 Department of Urology, University Hospital Hamburg-Eppendorf, Hamburg, Germany

**Keywords:** Radiation, Radical prostatectomy, Survival, Overtreatment, External beam radiotherapy, Adjuvant therapy, Salvage therapy

## Abstract

**Purpose:**

To test the association between external beam radiotherapy (EBRT) after radical prostatectomy (RP) vs RP only on rates of other-cause mortality (OCM) in men with prostate cancer (PCa).

**Patients and methods:**

Within the 2004–2016 Surveillance, Epidemiology, and End Results database, we identified 181,849 localized PCa patients, of whom 168,041 received RP only vs 13,808 who received RP + EBRT. Cumulative incidence plots displayed OCM between RP vs RP + EBRT after propensity score matching for age, PSA, clinical T- and N-stages, and biopsy Gleason scores. Multivariable competing risks regression models addressed OCM, accounting prostate cancer-specific mortality (CSM) as a competing event. Stratifications were made according to low- vs intermediate- vs high-risk groups and additionally according to age groups of ≤ 60, 61–70, and ≥ 71 years, within each risk group.

**Results:**

In low-, intermediate-, and high-risk patients, RP + EBRT rates were 2.7, 5.4 and 17.0%, respectively. After matching, 10-year OCM rates between RP and RP + EBRT were 7.7 vs 16.2% in low-, 9.4 vs 13.6% in intermediate-, and 11.4 vs 13.5% in high-risk patients (all *p* < 0.001), which, respectively, resulted in multivariable HR of 2.1, 1.3, and 1.2 (all *p* < 0.001). In subgroup analyses, excess OCM was recorded in low-risk RP + EBRT patients of all age groups (all *p* ≤ 0.03), but only in the older age group in intermediate-risk patients (61–70 years, *p* = 0.03) and finally, only in the oldest age group in high-risk patients (≥ 71 years, *p* = 0.02).

**Conclusion:**

Excess OCM was recorded in patients exposed to RT after RP. Its extent was most pronounced in low-risk patients, decreased in intermediate-risk patients, and was lowest in high-risk patients.

## Introduction

Adjuvant or salvage external beam radiotherapy therapy (EBRT) after radical prostatectomy (RP) for prostate cancer (PCa), with or without concomitant androgen deprivation therapy (ADT), is regarded as a valuable treatment option in highly select patients with adverse pathology at RP, such as positive surgical margins, Gleason score 8–10, ≥ pT3, or pN1 stage [1–5], and is recommended by the European Association of Urology, as well as by the National Comprehensive Cancer Network [6, 7]. Despite its cancer control advantage, EBRT after RP may cause toxicities. Potentially, some of long-term metabolic toxicities associated with EBRT and/or ADT may result in decreased life expectancy, due to mortality from non-PCa-related causes [8–14].

Therefore, to address this void, we tested other-cause mortality (OCM) in patients who received RP only vs RP + EBRT. We stratified the analyses according to low- vs intermediate- vs high-risk PCa, as well as according to age groups of ≤ 60, 61–70, and ≥ 70 years, within each risk group. We hypothesized that no OCM difference distinguish RP vs RP + EBRT, regardless of PCa risk group or age [9, 15].

## Materials and methods

### Study population

Within the Surveillance, Epidemiology, and End Results (SEER) database [16], we identified all patients with biopsy diagnosed, histologically confirmed adenocarcinoma of the prostate [International Classification of Disease for Oncology (ICD-O-3) code 8140 site code C61.9], who received treatment with either RP only or RP followed by EBRT (RP + EBRT), between 2004 and 2016. Autopsy or death certificate only cases were excluded. Eligibility criteria for the analyses were full available information on age at diagnosis, clinical T- and N-stages, PSA values, as well as biopsy Gleason scores. Metastatic PCa or patients aged 85 years or older were excluded. D’Amico low-risk group was defined as clinical T-stage ≤ 2a, Gleason sum ≤ 6, and PSA ≤ 10 ng/ml. D’Amico intermediate-risk group was defined as clinical T-stage 2b, Gleason sum 7, or PSA between 10 and 20 ng/ml. D’Amico high-risk group was defined as clinical T-stage ≥ T2c, Gleason sum 8–10, or PSA ≥ 20 ng/ml [17].

### Statistical analyses

Statistical modeling relied on propensity score matching (PSM) as well as competing risks regression models (CRR), ultimately investigating the effect of RP vs RP + EBRT on OCM, which was defined as any death not related to PCa [18]. Statistical analyses consisted of three steps. First, we stratified the population according to D’Amico low- vs intermediate- vs high-risk groups. RP + EBRT patients were matched to RP patients, within each risk group. PSM variables consisted of age at diagnosis (one-year intervals), clinical T-stage (exact), clinical N-stage (exact), PSA (1 ng/ml intervals), and biopsy Gleason score (exact).

Second, cumulative incidence and CRR models addressed OCM, after adjustment for prostate cancer-specific mortality (CSM). OCM rates and 95% confidence intervals (95% CI) were derived from the respective cumulative incidence functions. Within CRR models, further multivariable adjustment for socioeconomic status (distributed in quartiles), pathological Gleason score, and age categories of ≤ 60, 61–70, and ≥ 71 years was performed, ultimately providing hazard ratios (HR) and 95% CI.

Third, within each risk group (low- vs intermediate- vs high), we also stratified OCM analyses after separate PSM within age groups of ≤ 60, 61–70, and ≥ 71 years to test for effect modification according to age strata. Specifically, PSM was applied within each of the resulting nine risk- and age groups combinations: (1) low-risk, ≤ 60 years, (2) low-risk, 61–70 years, (3) low-risk, ≥ 71 years, (4) intermediate-risk, ≤ 60 years, (5) intermediate-risk, 61–70 years, (6) intermediate-risk, ≥ 71 years, (7) high-risk, ≤ 60 years, (8) high-risk, 61–70 years, and (9) high-risk, ≥ 71 years. Finally, CRR analyses were refitted within each of the nine groups. Censoring occurred when the patients were lost to follow-up or in case no event was recorded during study follow-up. All tests were two sided with a level of significance set at *p* < 0.05 and R software environment for statistical computing and graphics (version 3.4.3) was used for all analyses [19].

## Results

Of 181,849 eligible patients, 23.6% were low-risk, 51.8% were intermediate-risk, and 24.5% were high-risk. In general, RP + EBRT patients exhibited more unfavorable patient and tumor characteristics compared to RP patients, as evidenced by higher patient age, higher rates of more aggressive biopsy Gleason score, higher clinical T-stages, and higher rates of cN1 disease (Table 1). Median follow-up was 79 months (IQR 46-112) in the overall cohort, with 11,698 RP patients and 2,110 RP + EBRT patients at risk at 120 months.

**Table 1:** Patient characteristics of 181,849 localized prostate cancer patients within the 2004–2016 surveillance, epidemiology, and end results database

*n* = 181,849	D’Amico low-risk PCa (*n* = 42,951/23.6%)		D’Amico intermediate-risk PCa (*n* = 94,279/51.8%)		D’Amico high-risk PCa (*n* = 44,619/24.5%)	
	RP only (*n* = 41,811/97.3%)	RP + EBRT (*n* = 1,140/2.7%)	RP only (*n* = 89,195/94.6%)	RP + EBRT (*n* = 5,084/5.4%)	RP only (*n* = 37,035/83.0%)	RP + EBRT (*n* = 7,584/17.0%)
Age, years Median (IQR)	59 (55–64)	64 (58–69)	61 (56–66)	63 (57–68)	63 (57–67)	64 (58–69)
PSA, ng/ml median (IQR)	5.0 (4.1–6.3)	5.3 (4.0–6.9)	5.8 (4.5–8.2)	7.0 (5.0–10.7)	7.2 (5.0–14.1)	10.9 (6.1–24.5)
Follow-up, months median (IQR)	81 (55–115)	75 (46–110)	82 (48–112)	76 (43–107)	72 (33–109)	61 (27–98)
Socioeconomic status, n (%)						
1st quartile	9788 (23.4)	240 (21.1)	24,697 (28.0)	1285 (25.3)	9272 (25.0)	1994 (26.3)
2nd–3rd–4th quartile	32,023 (76.6)	900 (78.9)	64,228 (72.0)	3799 (74.4)	27,763 (75.0)	5590 (73.7)
Gleason Grade Group, *n* (%)						
I	41,811 (100)	1140 (100)	14,989 (16.8)	527 (10.4)	5575 (15.1)	308 (4.1)
II	–	–	50,630 (56.8)	2463 (48.4)	7402 (20.0)	861 (11.4)
III	–	–	16,197 (18.2)	1552 (30.5)	3389 (9.2)	781 (10.3)
IV	–	–	–	–	12,247 (33.1)	2430 (32.0)
V	–	–	–	–	7194 (19.4)	2927 (38.6)
Unknown	–	–	7379 (8.3)	542 (10.7)	1228 (3.3)	277 (3.7)
Clinical stage, *n* (%)						
cT1	38,621 (92.4)	1092 (95.8)	56,307 (63.1)	3417 (67.2)	12,061 (32.6)	2846 (37.5)
cT2	3190 (7.6)	48 (4.2)	32,888 (36.9)	1667 (32.8)	18,824 (50.8)	2823 (37.2)
cT3	–	–	–	–	3198 (8.6)	1139 (15.0)
cT4	–	–	–	–	172 (0.5)	218 (2.9)
cTx	–	–	–	–	2780 (7.5)	558 (7.4)
Nodal status, *n (%)*						
cN0	41,368 (98.9)	1114 (97.7)	87,485 (98.1)	4678 (92.0)	33,892 (91.5)	6053 (79.8)
cN1	72 (0.2)	12 (1.1)	1106 (1.2)	354 (7.0)	2821 (7.6)	1456 (19.2)
cNX	371 (0.9)	14 (1.2)	604 (0.7)	52 (1.0)	322 (0.9)	75 (1.0)

Stratification was performed according to D’Amico low-, intermediate-, and high-risk groups as well as treatment type (radical prostatectomy alone vs radical prostatectomy and adjuvant or salvage external beam radiotherapy

*RP* radical prostatectomy, *EBRT* external beam radiotherapy, *PSA* prostate-specific antigen, *PCa* prostate cancer

### RP vs RP + EBRT in low-risk prostate cancer

**Fig. 1:**
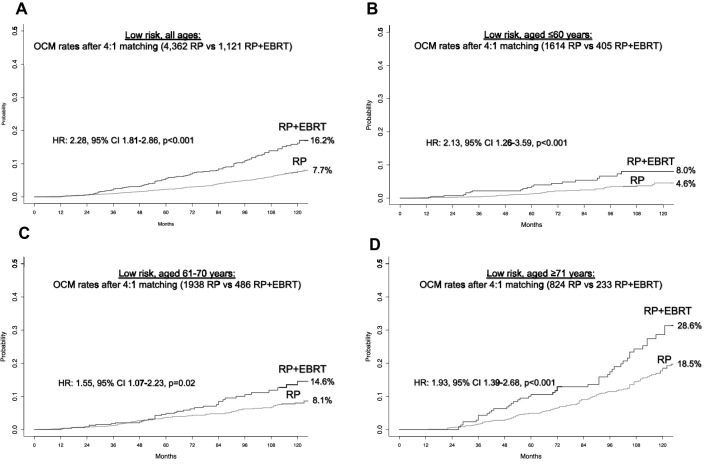
Cumulative incidence plots displaying differences in 10-year other-cause mortality (OCM) rates between D’Amico low-risk prostate cancer patients treated with radical prostatectomy (RP) vs patients treated with RP and external beam radiotherapy (RP + EBRT), after 4:1 propensity score matching for patient and tumor characteristics. Panel **A** displays low-risk patients of all ages, Panels **B**–**D** display low-risk patients according to age groups strata: ≤ 60 years, 61–70 years, and ≥ 71 years

In the 42,951 low-risk patients, the rate of RP + EBRT was 2.7%. PSM focused on 1,121 RP + EBRT patients, who were matched with four RP controls (*n* = 4,326). After PSM, no residual difference in patient and/or tumor characteristics remained (all *p* ≥ 0.6). After PSM, 10-year OCM rates (and 95% CI) were 16.2% (13.1–19.6%) vs 7.7% (6.5–8.9%) for RP + EBRT vs RP, respectively (Fig. 1A). In multivariable CRR analyses (Table 2), RP + EBRT was an independent predictor of higher OCM compared to RP (HR 2.1, 95% CI 1.7–2.6, *p* < 0.001). Within the same multivariable CRR models, compared to the reference of patients aged ≤ 60 years, 61–70-year-old patients as well as ≥ 71-year-old patients also exhibited higher OCM (HR 2.3 for 61–70 years and HR 4.2 for ≥ 71 years, both *p* < 0.001, (Table 2).

**Table 2:** Multivariable competing risks regression models predicting other-cause mortality in propensity score-matched D’Amico low-, intermediate-, and high-risk populations according to treatment status [radical prostatectomy + external beam radiotherapy (RP + EBRT) vs RP alone, while RP alone was used as reference] and according to age groups (≤ 50, 51–60, 61–70, and ≥ 71 years; while the largest subgroup of patients aged 61–70 years was used as reference)

Variables	Low-risk group			Intermediate-risk group			High-risk group		
	HR	95% CI	*p* value	HR	95% CI	*p* value	HR	95% CI	*p* value
RP + EBRT (vs RP only)	2.1	1.7–2.6	< 0.001	1.3	1.2–1.5	< 0.001	1.2	1.1–1.4	< 0.001
≤ 60 years (Ref.)	1.0	–	–	1.0	–	–	1.0	–	–
61–70 years	2.3	1.6–3.3	< 0.001	1.9	1.6–2.1	< 0.001	1.8	1.5–2.1	< 0.001
≥ 71 years	4.2	2.5–7.0	< 0.001	4.1	3.6–4.8	< 0.001	4.1	3.5–4.9	< 0.001

*HR* Hazard ratio, *CI* Confidence interval

Subsequent stratifications according to age groups identified 409, 489, and 242 RP + EBRT patients in age groups of ≤ 60, 61–70, and ≥ 71 years, respectively. These were matched with four RP controls within each respective age group and yielded cohorts of 405 RP + EBRT vs 1,614 RP patients in the age group ≤ 60 years, 486 RP + EBRT vs 1,938 RP patients in the age group 61–70 years and 233 RP + EBRT vs 824 RP patients in the age group ≥ 71 years. After PSM, no residual differences in patient and/or tumor characteristics in each comparison remained within each age stratum (all *p* ≥ 0.8). After PSM, OCM rates (and 95% CI) between RP + EBRT vs RP were 8.0% (4.9–12.2%) vs 4.6% (3.1–6.4%) in the age group ≤ 60 years, 14.6% (10.1–19.9%) vs 8.1% (6.4–10.0%) in the age group 61–70 years, and 28.6% (21.1–36.6%) vs 18.5% (15.0–22.3%) in the age group ≥ 71 years, respectively (Fig. 1B, C, D). These OCM rates translated into a multivariable CRR HR of 2.2, 95% CI 1.3–3.6, *p* < 0.001 for ≤ 60 years, HR of 1.5, 95% CI 1.1–2.2, *p* = 0.03 for 61–70 years, and HR of 1.9, 95% CI 1.4–2.6, *p* < 0.001 for ≥ 71 years.

### RP vs RP + EBRT in intermediate-risk prostate cancer

**Fig. 2:**
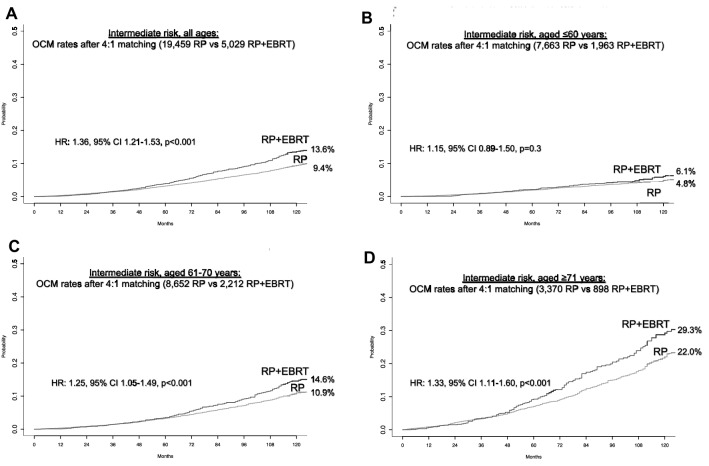
Cumulative incidence plots displaying differences in 10-year other-cause mortality (OCM) rates between D’Amico intermediate-risk prostate cancer patients treated with radical prostatectomy (RP) vs patients treated with RP and external beam radiotherapy (RP + EBRT), after 4:1 propensity score matching for patient and tumor characteristics. Panel **A** displays intermediate-risk patients of all ages. Panels **B**–**D** display low-risk patients according to age groups strata: ≤ 60 years, 61–70 years, and ≥ 71 years

In 94,279 intermediate-risk patients, the rate of RP + EBRT was 5.4%. PSM focused on 5,029 RP + EBRT patients, who were matched with four RP controls (*n* = 19,459). After PSM, no residual difference in patient and/or tumor characteristics remained (all *p* ≥ 0.6). After PSM, 10-year OCM rates (and 95% CI) were 13.6% (12.2–15.1%) vs 9.4% (8.8–10.0%) for RP + EBRT vs RP, respectively (Fig. 2A). In multivariable CRR analyses (Table 2), RP + EBRT was an independent predictor of higher OCM compared to RP (HR 1.3, 95% CI 1.2–1.5, *p* < 0.001). Within the same multivariable CRR models, compared to the reference of patients aged ≤ 60 years, both 61–70-year-old patients as well as ≥ 71-year-old patients also exhibited higher OCM (HR 1.9 for 61–70 years and HR 4.1 for ≥ 71 years, both *p* < 0.001, Table 2).

Subsequent stratifications according to age groups identified 1,969, 2,219, and 896 RP + EBRT patients in age groups of ≤ 60, 61–70, and ≥ 71 years, respectively. These were matched with four RP controls within each respective age group and yielded cohorts of 1,963 RP + EBRT vs 7,663 RP patients in the age group ≤ 60 years, 2,212 RP + EBRT vs 8,652 RP patients in the age group 61–70 years, and 898 RP + EBRT vs 3,370 RP patients in the age group ≥ 71 years. After PSM, no residual differences in patient and/or tumor characteristics in each comparison remained within each age stratum (all *p* ≥ 0.8). In the matched cohorts, OCM rates (and 95% CI) between RP + EBRT vs RP were 6.1% (4.6–7.7%) vs 4.8% (4.2–5.6%) in the age group ≤ 60 years, 14.6% (12.3–17.0%) vs 10.9% (9.9–12.0%) in the age group 61–70 years, and 29.3% (24.7–34.0%) vs 22.0% (19.8–24.4%) in the age group ≥ 71 years, respectively (Fig. 2B, C, D). These OCM rates translated into a multivariable CRR HR of 1.1, 95% CI 0.9–1.5, *p* = 0.3 for ≤ 60 years, HR of 1.2, 95% CI 1.0–1.5, *p* = 0.03 for 61–70 years, and HR of 1.2, 95% CI 1.0–1.4 *p* = 0.07 for ≥ 71 years.

### RP vs RP + EBRT in high-risk prostate cancer

**Fig. 3:**
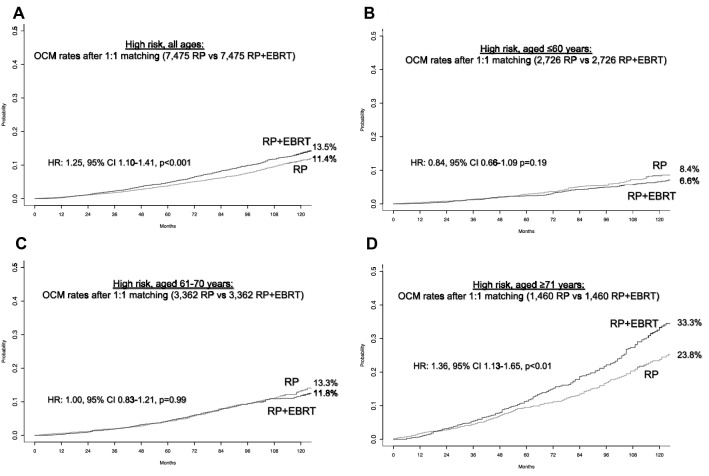
Cumulative incidence plots displaying differences in 10-year other-cause mortality (OCM) rates between D’Amico high-risk prostate cancer patients treated with radical prostatectomy (RP) vs patients treated with RP and external beam radiotherapy (RP + EBRT), after 4:1 propensity score matching for patient and tumor characteristics. Panel **A** displays high-risk patients of all ages. Panels **B**–**D** display low-risk patients according to age groups strata: ≤ 60 years, 61–70 years, ≥ 71 years

In 44,619 high-risk patients, the rate of RP + EBRT was 17.0%. PSM focused on 7,475 RP + EBRT patients, who were matched with one RP control (*n* = 7,475). After PSM, no residual difference in patient and/or tumor characteristics remained (all *p* ≥ 0.7). After PSM, 10-year OCM rates (and 95% CI) were 13.5% (12.4–14.8) vs 11.4% (10.3–12.6) for RP + EBRT vs RP, respectively (Fig. 3A). In multivariable CRR analyses (Table 2), RP + EBRT was an independent predictor of higher OCM compared to RP (HR 1.2, 95% CI 1.1–1.4, *p* < 0.001). Within the same multivariable CRR models, compared to the reference of patients aged ≤ 60 years, both 61–70-year-old patients as well as ≥ 71-year-old patients also exhibited higher OCM (HR 1.8 for 61–70 years and HR 4.1 for ≥ 71 years, both *p* < 0.001, Table 2).

Subsequent stratifications according to age groups identified 2,735, 3,366, and 1,483 RP + EBRT patients in age groups of ≤ 60, 61–70, and ≥ 71 years, respectively. These were matched with one RP control within each respective age group and yielded cohorts of 2,726 RP + EBRT vs 2,726 RP patients in the age group ≤ 60 years, 3,362 RP + EBRT vs 3,362 RP patients in the age group 61–70 years, and 1,460 RP + EBRT vs 1,460 RP patients in the age group ≥ 71 years. After PSM, no residual differences in patient and tumor characteristics in each comparison remained within each age stratum (all *p* ≥ 0.8). In the matched cohorts, OCM rates (and 95% CI) between RP + EBRT vs RP were 6.6% (5.3–8.0%) vs 8.4% (6.9–10.0%) for ≤ 60 years, 11.8% (10.2–13.6%) vs 13.3% (11.5–15.3%) for 61–70 years, and 33.3% (29.2–37.4%) vs 23.8% (20.3–27.4%) for ≥ 71 years, respectively (Fig. 3B, C, D). These OCM rates translated into a multivariable CRR HR of 0.8, 95% CI 0.7–1.1, *p* = 0.18 for ≤ 60 years, HR of 1.0, 95% CI 0.8–1.2, *p* = 0.9 for 61–70 years, and HR of 1.3, 95% CI 1.0–1.5, *p* = 0.02 for ≥ 71 years.

## Discussion

EBRT after RP is used in up to 50% of patients with adverse pathology [20, 21]. Despite its benefits on cancer control, the effect of EBRT after RP on OCM is unknown. We addressed this void and tested OCM rates between RP only vs matched RP + EBRT patients. We hypothesized that no OCM rate difference should exist between these two groups, since combination therapy candidates have been selected as surgical candidates initially and therefore it may be postulated that their OCM should be very similar to that of their RP only counterparts. Our analyses yielded several noteworthy observations.

First, the rate of RP + EBRT ranged from 2.7% in the low-risk group, 5.4% in the intermediate-risk group, and to 17.0% in the high-risk group. Therefore, non-negligible proportions of intermediate-risk and high-risk patients are exposed to EBRT after RP. Unfortunately, our data did not allow to explain the underlying rationale for EBRT after RP in these individuals, due to insufficiently detailed data regarding RP pathology and/or surgical margins.

Second, important differences in patient, as well as tumor characteristics between RP and RP + EBRT patients were recorded, across all risk strata. Specifically, RP + EBRT patients exhibited higher median patient age, higher median PSA values, higher cT stages, higher biopsy Gleason score, and higher rates of clinically node-positive disease. These differences demonstrate the need for strictest adjustment in the form of PSM and additional multivariable adjustment, as well as adjustment for CSM, to ensure that RP and RP + EBRT populations are comparable regarding patient and tumor characteristics.

Third, in matched competing risks analyses, we invariably recorded higher OCM in RP + EBRT than RP only patients. The excess OCM after RP + EBRT ranged from highest in the low-risk group (+ 8.5%), to intermediate in the intermediate-risk group (+ 4.2%), and to lowest in the high-risk group (+ 2.1%). All of the above OCM rate differences achieved independent predictor status despite the strictest PSM, multivariable adjustment and additional adjustment for CSM and respectively yielded multivariable CRR HR of 2.1 (*p* < 0.001) in low-risk PCa, HR of 1.3 (*p* < 0.001) in intermediate-risk PCa, and HR of 1.2 (*p* < 0.001) in high-risk PCa. Additionally, we tested for effect modification according to age strata. Here, we identified important effect modifications of OCM that increased with age. The effect was most pronounced in oldest patients (≥ 71 years), across all risk strata (HR 4.2 in low-risk, HR 4.1 in intermediate-risk, and HR 4.1 in high-risk, all *p* < 0.001). Its size was intermediate in the intermediate age category (61–70 years), also across all risk strata (HR of 2.3 in low-risk, HR of 1.9 in intermediate-risk, and HR of 1.8 in high-risk, all *p* < 0.001).

The above findings indicate that the effect of EBRT after RP is most pronounced in elderly patients (≥ 71 years) and intermediate in the intermediate age strata (61–70 years). Interestingly, this effect is of similar relative magnitude in all risk groups. However, its absolute magnitude, expressed in absolute OCM rate differences, is strongest in low-risk PCa groups. This observation may partly be explained by competing CSM that mostly affects high-risk patients and is least operational in low-risk patients. Based on the highest absolute rate of excess OCM after RP + EBRT in low-risk patients and lowest absolute rate of excess OCM after RP + EBRT in high-risk patients, it is unlikely that ADT may represent an underlying cause, since the opposite association would be expected, if ADT was directly related to OCM rates. Nonetheless, an interplay between EBRT, ADT, and patient characteristics, including age, must be suspected. More detailed, ideally prospective studies, will allow to validate our observations and elucidate the true causative factors. Additionally, our observations question the selection criteria for EBRT after RP that predominantly target elderly individuals. Ideally, intensification of therapy should predominantly focus on younger patients.

Taken together, we recorded excess OCM after EBRT delivered to RP patients, relative to their counterparts treated with RP only. The excess OCM was operational across all risk strata and ranged from + 2.1% (high-risk), + 4.2% (intermediate-risk), to + 8.5% (low-risk). Interestingly, within each PCa risk stratum, intermediate age (61–70 years) predisposed to two-fold OCM increase and oldest age (≥ 71 years) predisposed to four-fold OCM increase. Nonetheless, the absolute increase in OCM was highly statistically significant even in the high-risk group and even despite strictest PSM, multivariable adjustment and accounting for CSM as a competing event in CRR. In consequence, our observations deserve further investigation in other epidemiological and/or institutional databases to validate our findings and to elucidate the underlying causes.

Our study has limitations and should be interpreted in the context of its retrospective and population-based design. First, no distinction could be made according to adjuvant or salvage EBRT after RP. Second, no information on the type or dose of EBRT or the type or dose of concomitant ADT was available. Third, the true cause of death in patients dying of OCM is not incorporated in SEER and therefore a direct association with the toxic effect of radiation and/or ADT can also not be drawn. Finally, no information was available about cancer control outcomes that preceded OCM or CSM. However, since the study was focused on OCM rates that were adjusted for CSM in competing risks analyses, this limitation does not affect its primary outcome.

## Conclusion

Excess OCM was recorded in patients exposed to RT after RP. Its extent was most pronounced in low-risk patients, decreased in intermediate-risk patients, and was lowest in high-risk patients.

## Data Availability

R software environment for statistical computing and graphics (version 3.4.0 for MAC OS X; http://www.r-project.org/) was used for all statistical analyses. Used codes for analyses can be provided. The data that support the findings of this study are available from the Surveillance, Epidemiology, and End results database, (SEER) but restrictions apply to the availability of these data, which were used under license for the current study and so are not publicly available. Data are, however, available from the authors upon reasonable request and with permission of SEER.
